# Physical and Biological Performance Evaluation of Disinfection Systems for Transportation Vehicles against AI Virus

**DOI:** 10.4014/jmb.2103.03024

**Published:** 2021-06-01

**Authors:** Hansung Chung, Kwanghoon Choi, Sungkwan Kim, Sukwon Kim, Kyungwoo Lee, Nonghoon Choe

**Affiliations:** 1 College of Veterinary Medicine, Konkuk University, Seoul 05029, Republic of Korea; 2 Department of Animal Science and Technology, Konkuk University, Seoul 05029, Republic of Korea

**Keywords:** Disinfection facility, disinfectant solution coverage, viral reduction, performance evaluation method, biosecurity

## Abstract

To prevent the outbreak of infectious diseases that inflict huge economic and social losses, domestic livestock farms and related facilities have introduced automatic and semiautomatic disinfectant solution-spraying systems for vehicles. However, the facility standards and specifications vary by manufacturer, and no scientific performance evaluation has been conducted. The puropose of this study is to develop physical and biological evaluation methods. Physical and biological appraisals were conducted using two types of disinfection facilities (tunnel- and U-type) and two types of vehicles (passenger car, truck). Water-sensitive paper was used to evaluate the physical performance values for the disinfection facilities. In addition, to assess their biological performance, carriers containing low-pathogenic avian influenza virus were attached to vehicles, and the viral reduction was measured after the vehicles moved through the facility. The tunnel-type had rates of coverage in the range of 70–90% for the passenger car and 60–90% for the truck. At least 4-log virus reduction after spraying for 1–5 min was shown for both vehicles. For the U-type facility evaluation, the coverage rates were in the range of 60–90% for the passenger car and at least 90% for the truck. More than 4-log viral reduction was estimated within a spraying time of 5 min. To reduce viruses on the surface of vehicles by at least 4 log within a short period, the disinfectant solution should cover at least 71% of the pathogens. In conclusion, we were able to assess the physical and biological performance criteria for disinfection facilities aboard transportation vehicles.

## Introduction

Foot-and-mouth disease (FMD) and highly pathogenic avian influenza (HPAI) fall under class 1 livestock infectious diseases that show high contagiousness and the rapid appearance of clinical symptoms. The infection pathways of FMD virus (FMDV) and avian influenza virus (AIV) can be largely divided into ‘direct contact infection,’ occurring due to direct contact with the excretions, droplet air, and saliva of infected animals, and ‘indirect infection’ caused by people (livestock workers and veterinarians), and contaminated vehicles (*e.g.*, feed transporting, live domestic animal transporting, and consulting vehicles), clothing, feed, apparatuses, and equipment [1-3]. The viruses may be spread by vectors such as rats, birds, and insects that have come into contact with domestic animals’ secretions or excretions in the neighboring areas or by wild migratory birds or contaminated livestock products [4-9]. Since 2000, outbreaks of FMD and HPAI have been continuing, leading to the culling of infected livestock with the investment of huge amounts of human and material resources. In particular, during an outbreak of FMD that occurred in October 2010, 3,479,962 animals were buried at 6,241 farms nationwide at a cost of 2.7 trillion Korean won (KRW). Due to an outbreak of HPAI that occurred in November 2016, 38,064 poultry were buried at 1,131 farms nationwide at a cost of over 300 billion KRW [10-12].

A common feature of these FMD and avian influenza (AI) outbreaks was that they spread when the surfaces of apparatus, people, and objects come into contact with organisms contaminated with pathogens and are then moved, without being properly disinfected. Domestic and overseas studies have reported vehicles, carrying devices, and drivers, as major causes of virus introduction into livestock farms and it is known that appropriate cleaning and disinfection of accessing people, vehicles, articles, and equipment are important [13-21]. With regard to the possibility of virus introduction through vehicles, Lowe *et al*. [18] observed increased virus counts on the surface of transport vehicles after livestock loading. Through epidemiological analysis, Wee *et al*. [22] and Vander Waal *et al*. [23] identified vehicles as a major cause of the spread of diseases, and that vehicle standstill was effective in preventing the spread of infectious diseases [17, 24]. It has been documented that during the FMD outbreak in South Korea in 2010, vehicle driving restrictions due to heavy snowfall were helpful in minimizing the spread of the disease [25].

Among the total of 152 farms in South Korea where FMD occurred in 2010–2011, 105 were associated with vehicles for transportation of veterinarians, feed, and livestock (14 cases). The FMD outbreak in 2014–2015 involved 185 farms, of which, 78.9% (143 cases) and 10.8% (23 cases) were assumed to have been infected by viruses introduced through vehicles and people, respectively. The analysis results indicated that FMDV contamination of vehicles and people was mainly associated with visits to stock-raising facilities (50.3%: 93 cases) and slaughterhouses (40.5%: 75 cases) [10]. Among the 419 farms contaminated with HPAI in 2016–2017, 214 (51.1%) were assumed to have been infected by viruses introduced by farm-accessing vehicles [10, 25, 26].

To prevent the introduction and outbreak of infectious diseases (*e.g.*, FMD, HPAI) that cause huge economic and social losses, most domestic livestock farms and related facilities in the U.S. are introducing disinfectant spraying facilities. However, those disinfection facilities are supplied in the states where facility standards and specifications differ among the disinfection facility manufacturers, and performance tests are not scientifically conducted.

Previous studies related to disinfection facilities have been limited to functional performance, such as nozzle discharge rates, nozzle positions, and spray ratios, of mechanical disinfectors [27, 28] or spray temperatures and application ratios as a function of pressure level [25, 29]. Dee *et al*. [30, 31] and Patterson *et al*. [32] determined the degree of viral reduction on transport vehicles contaminated with porcine respiratory and reproductive syndrome virus (PRRSV) and porcine circovirus (PCV), respectively, after passing through a disinfection facility. However, such studies conducted disinfection for a set time (10 to 90 min) using trailers reduced in size. Domestically, it seems that these methods are not being applied because vehicles accessing local stock-raising facilities are not disinfected for more than 1 min at the maximum. That is, no study has evaluated pathogen extinction and inactivation in a short time using actual vehicles and disinfection facilities.

In the present study, we sought to identify appropriate disinfection facility operation methods (appropriate disinfection time) through the measurement of disinfectant solution coverage for the vehicle surface and the values for viral reduction after a vehicle has passed through the disinfection facility. We also examined whether the virus removal (biological) ability can be indirectly evaluated by measuring the coverage ratios (physical).

## Materials and Methods

### Disinfection Facility Specification

The disinfection facilities used in this study are vehicle disinfection facilities that spray a disinfectant solution made by diluting a disinfectant with water at a certain ratio. One of the facilities is a tunnel-type (12 m × 4.5 m × 5 m, length (L) × width (W) × height (H)) and the other is a wall-type (U-type; 2 m × 4.5 m × 2.8 m, L × W × H). The wall-type disinfection facility was installed with 7 nozzles at the bottom and 20 nozzles on the sides, whereas the tunnel-type disinfection facility was installed with 42 nozzles at the bottom and 160 nozzles on the sides.

The tunnel-type disinfection facility allows setting of disinfectant solution spray times and was designed and fabricated to have vehicle recognition sensors and speed shutters installed at the access road so that vehicle access can be managed. The operation sequence is as follows: first, the entrance shutter is opened when a vehicle is entering; second, the entrance shutter is closed, and the spray of the disinfectant solution starts when the vehicle stops after entering the facility; finally, the entrance shutter is opened, and the vehicle leaves when the spray of the disinfectant solution has finished.

The wall-type disinfection facility is automatically operated through the sensor when a vehicle enters. The operation sequence is as follows: first, the spray of the disinfectant solution starts when the vehicle enters; second, the spray of the disinfectant solution is terminated after the vehicle passes through the disinfection facility at low speeds (3–5 km/h).

The disinfectant used was a product permitted for commercial sale by the Animal and Plant Quarantine Agency in Korea, and the main ingredient was citric acid. The container of the disinfectant was opened on the day of the experiment, and the disinfectant was diluted with water, pursuant to the manufacturer’s instructions (recommended concentration 1:250) before being used.

### Vehicle Specification

Most of the vehicles that access stock-raising facilities (*e.g.*, livestock farms, slaughterhouses, poultry slaughterhouses, feed factories) for transportation of live domestic animals, feed, and manure, for example, are large trucks (5 tons). Vehicles that access the facilities for medical treatment, vaccination, artificial insemination, consulting, sampling, and disinfection are passenger vehicles or 1-ton trucks. In the present study, a passenger vehicle (4,820 mm × 1,835 mm × 1,470 mm, L × W × H) and a 5-ton truck (8,660 mm × 2,420mm × 3,060 mm, L × W × H) were used.

### Identification of Disinfectant Solution Application Performance

Specially-coated, yellow water-sensitive papers (WSP) (76 × 52 mm^2^, 20301, TeeJet Technologies, USA), which turn dark blue on contact with water, were used to ascertain the disinfectant solution physically covering the vehicle surface passing through the disinfection facility.

For the assessment, vehicles were divided into four sections (front, back, bottom, and side), and numerous WSPs were attached to the bottom, where most organic matter, such as soil feces, were expected to be present, and to the sides, which frequently come into contact with passengers. Table 1 lists the locations and quantity of the WSPs that were attached. The WSPs were attached after being inserted into Petri dishes (15 × 90 mm, SPL, Korea) so that the disinfectant solution flowing down the vehicle surface and the WSPs would not additionally react.

When the vehicle had stopped in the tunnel-type disinfection facility, the disinfectant solution was sprayed for 30 and 60 s, respectively, and the application performances by region were determined. After a vehicle passed through the wall-type disinfection facility, in which the disinfectant solution was being sprayed at speeds of 3–5km h^-1^, the application performances by region were measured. All processes were repeated twice.

### Image Analysis

After a vehicle passed through the disinfection facility, the WSPs that reacted with the disinfectant solution were collected from the vehicle and photographed (EOS750D, Canon, Japan). The “analyze particle” function of Collins [33] and Ferreira and Rasband [34] and the “Threshold” method of Jensen [35] were used with the image analysis software ImageJ (ver. 1.60, NIH, USA), to measure the area and ratio of discolored WSPs. The steps for using the “analyze particle” function are as follows. First, select only the subject area of the pictures taken with the "Crop" function. Second, adjust brightness and contrast using “Brightness/Contrast”. Finally, when using “Type/8-bit” to convert the image to 256 shades (8-bit) of gray, the image was changed to black and white (binary). When “Threshold” was used to measure areas, the black portion in binary image became red areas. The red areas were measured as result data.

### Virus Preparation

Low pathogenic AIV (A/chicken/Korea/MS96/1996) was used. The virus was inoculated into the allantoic fluid of 10-day-old chicken embryonic eggs and incubated at 37°C for 72 h. After incubation, the allantoic fluid was chilled at 4°C for 3 h and then harvested. The harvested allantoic fluid was centrifuged (907 g, 4°C, 20 min) to remove solid components. The chicken embryo infectious doses per milliliter (EID_50_/ml^-1^) of the allantoic fluid containing the virus was identified through hemagglutination tests, conducted according to the method of Kang [36] and Jang *et al*. [37], based on the Office International des Epizooties (OIE) standards [38]and Spearman–Kärber method. The experiment was repeated twice. The virus used was ensured to be at least 7.5 log and kept at –70°C until used.

### Carrier Pretreatment and Attachment Method

Round stainless-steel carriers (AISI 304, Posco, Korea) with a diameter of 2 cm and 1.5–1.8 mm (H) were used after washing with sterilized deionized water twice and high-pressure sterilization (121°C, 15 min).

The carrier attachment positions and the quantity of carriers on the vehicle surface were determined mainly among parts where the majority of organic matter, such as soil and feces, were expected to be present or parts that frequently come into contact with vehicle passengers. The carriers were put into Petri dishes (15 × 90 mm) before being attached to the vehicle so that the virus on the carrier would not additionally react with the disinfectant solution flowing down the vehicle surface.

### Biological Performance Evaluation Method

The virus culture medium and 20% FBS (Fetal Bovine Serum) were mixed in equal amounts, and 100 µl of the mixture was inoculated onto the carriers. The inoculated carriers were placed in 6-well plates (Nunc, Denmark) and dried on a clean bench for 50–55 min. The dried carriers were attached to the designated positions on the experimental vehicle. The disinfectant solution was sprayed and reacted with the virus on the carrier while the experimental vehicle was passing through the disinfection facility. The tunnel-type disinfection facility sprayed the disinfectant solution for 30 s after the vehicle stopped inside the facility. The wall-type disinfection facility sprayed the disinfectant solution while the vehicle was passing through the disinfection facility at speeds of 3–5km/h.

The reaction times between the virus on the carriers and the disinfectant solution were set to 1, 5, and 10 min, respectively, immediately after the vehicle passed through the facility. After the reaction time, each carrier was recovered into a 50 ml conical tube containing 10 ml of neutralization medium (20% FBS), and vortexed for 3 min.

### Identifying the Degree of Viral Reduction

Decimal serial dilutions of the mixture of the neutralization medium and the virus were prepared to 10^5^ using PBS (Sigma–Aldrich) that had been prefiltered (0.45 µm). After inoculating 200 µl of the diluted solution into each of five, 10-day-old chicken embryonic eggs, the eggs were incubated at 37°C for 4 days. Dead chicken embryonic eggs were discarded on the first day of incubation, through candling. After the incubation, the EID_50_/ml^-1^ of the allantoic fluid containing the virus was identified through hemagglutination tests and the Spearman–Kärber method, as described by Kang [36] and Jang *et al*. [37], based on the OIE [38] standard. All experiments were repeated twice.

Since the evaluation criteria for the efficacy of domestic disinfection facilities on the virus were not prepared, the evaluation criteria in the guidelines for disinfectant efficacy tests of the Animal and Plant Quarantine Agency that require pathogens to become extinct or inactivated at least to 10^4^ ml^-1^ were applied.

### Statistical Analysis

Data analysis was performed using GraphPad Prism 5 software (ver. 5.01, GraphPad, Inc., USA). All data were expressed as mean and standard deviation. The means were analyzed using ANOVA followed by a Tukey test (*p* < 0.05). A regression equation was used to determine the relationship between the vehicle surface disinfectant solution coverage ratios and the viral reduction values. The regression equation obtained was a simple linear regression equation using the vehicle surface coverage ratios (%) as the independent variable (X) and the AI viral reduction values as the dependent variable (Y). The significance was tested by Pearson’s analysis, and the coefficient of determination (R^2^) was determined. Finally, the results of the physical (WSP discoloration rate) and biological performance evaluation (viral reduction values on the carrier) obtained from the same locations on the vehicle after passing through the disinfection facility were selected.

## Results

### Disinfectant Solution Coverage Evaluation

**Tunnel-type disinfection facility**. After the passenger vehicle and the truck stopped in the tunnel-type disinfection facility, the disinfectant solution was sprayed for 30 and 60 s, respectively, and changes in the color of the WSPs attached to the front, rear, bottom, and sides of the vehicles were measured.

**Fig. 1:**
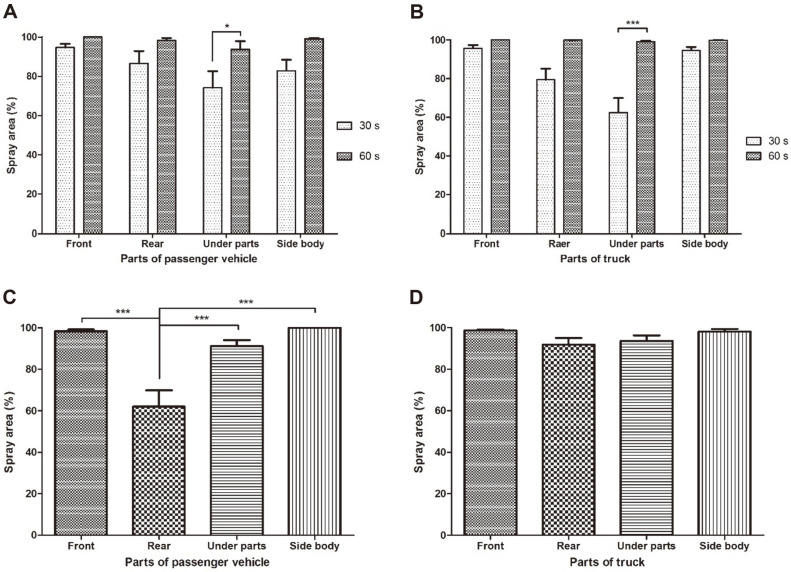
Spray performance of the disinfection facilities using water-sensitive paper (WSP). (**A**) Tunnel-type facility tested on passenger vehicle sprayed with disinfectant for 30 and 60 s. (**B**) Tunnel-type facility tested on truck sprayed with disinfectant for 30 and 60 s. (**C**) U-type facility tested on passenger vehicle passing through, at 3-5 km h^-1^. (**D**) U-type facility tested on truck passing through at 3-5 km h-1. *p < 0.05, **p < 0.005, ***p < 0.001.

When the disinfectant solution was sprayed on the passenger vehicle, disinfectant solution coverage ratios not lower than 70% were identified for each section. When the disinfectant solution was sprayed for 30 s, the disinfectant solution coverage ratios were front 94.65 ± 1.84%, rear 86.46 ± 6.39%, bottom 74.10 ± 8.45%, and sides 82.80 ± 5.66%. When the disinfectant solution was sprayed for 60 s, the disinfectant solution coverage ratios were front 100%, rear 98.33 ± 1.14%, bottom 93.78 ± 4.19%, and sides 99.15 ± 0.29%. Regarding the bottom of the passenger vehicle, the disinfectant solution coverage ratios were affected (*p* < 0.05) by the disinfectant solution spray times but no significant difference in the resultant coverage ratios could be identified between spray times at the front, rear, or sides (Fig. 1A).

When the disinfectant solution was sprayed on the large truck for 60 s, disinfectant solution coverage ratios not lower than 99% were identified for each section. The disinfectant solution coverage ratios were front 100%, rear 99.93 ± 0.04%, bottom 99.11 ± 0.42%, and sides 99.82 ± 0.05%. When the disinfectant solution was sprayed for 30 s, whereas coverage ratios not lower than 90% were identified at the front (95.58 ± 1.66%) and sides (94.52 ± 1.70%), relatively low values were shown at the rear (79.35 ± 5.76%) and bottom (62.37 ± 7.51%). Whereas similar coverage ratios at the front, sides, or rear of the large truck were identified for both spray times (*p* > 0.05), the spray times markedly influenced the coverage ratios on the bottom (*p* < 0.001) (Fig. 1B).

**Wall-type disinfection facility.** The passenger vehicle and large truck passed the wall-type disinfection facility that was spraying the disinfectant solution at speeds of 3–5 km/h, and disinfectant solution coverage values were measured at the front, rear, bottom, and sides of the vehicles.

The resultant disinfectant solution coverage ratios at the passenger vehicle surface were at least 90% at all sections (front 98.33 ± 0.82%, bottom 91.18 ± 2.85%, sides 100%), except for the rear (61.98 ± 7.91%), which was considerably different (*p* < 0.001) compared to the values at the front, bottom, and sides (Fig. 1C).

For the large truck, coverage values not lower than 90% were identified for all sections (Fig. 1D): front 98.63 ± 0.45%, rear 91.86 ± 3.07%, bottom 93.56 ± 2.66%, and sides 98.12 ± 1.18%, and no significant difference could be seen between the sections (*p* > 0.05).

### AIV Reduction Evaluation

**Tunnel-type disinfection facility**. The disinfectant solution was sprayed (30 s) after the passenger vehicle and large truck stopped in the tunnel-type disinfection facility, and the viral reduction values were measured at 1, 5, and 10 min reaction times, immediately after the vehicles passed through the facility.

**Fig. 2:**
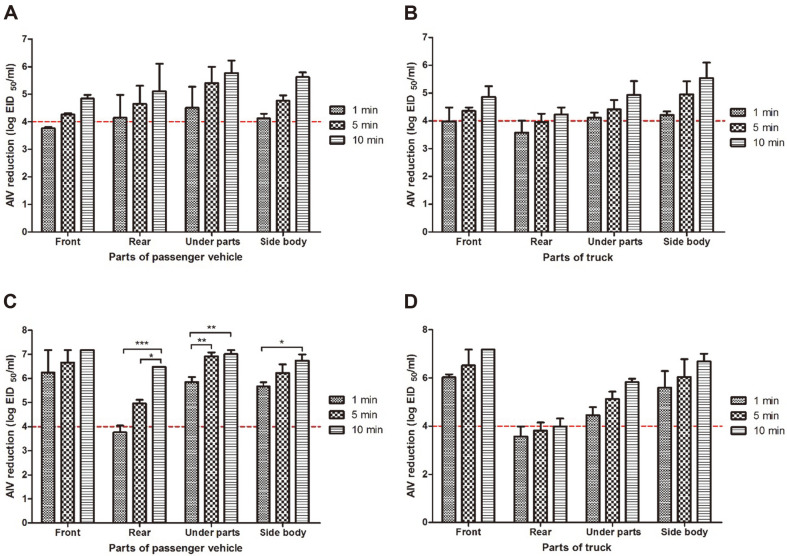
Avian influenza virus (AIV) reduction achieved by the disinfection facility. (**A**) Tunnel-type facility tested on passenger vehicle; disinfectant spray time: 30 s; treatment time: 1, 5, and 10 min. (**B**) Tunnel-type facility tested on truck; disinfectant spray time: 30 s; treatment time: 1, 5, and 10 min. (**C**) U-type facility tested on passenger vehicle; passing speed: 3- 5 km h-1; treatment time: 1, 5, and 10 min. (**D**) U-type facility tested on truck; passing speed: 3-5 km h-1; treatment time: 1, 5, and 10 min. *p < 0.05, **p < 0.005, ***p < 0.001.

Viral reductions of at least 4 log could be identified at the rear (4.15 ± 0.84 log), bottom (4.51 ± 0.76 log), and sides (4.12 ± 0.17 log) of the passenger vehicle, but not the vehicle front (3.76 ± 0.05 log) after reaction for 1 min. After reaction for 5 min, the degrees of viral reduction at the front, rear, bottom, and side parts were 4.27 ± 0.04, 4.65 ± 0.66, 5.41 ± 0.59, and 4.77 ± 0.20 log, respectively. After reaction for 10 min, reductions of at least 5 log were identified at the rear (5.11 ± 1.00 log), bottom (5.77 ± 0.46 log), and sides (5.62 ± 0.18 log), but not the front (4.85 ± 0.14 log). The viral reduction values by disinfectant solution spray time were not influenced by the evaluated locations on the passenger vehicle (*p* > 0.05) (Fig. 2A).

For the large truck, viral reductions of at least 4 log were observed after spraying for 1 and 5 min, respectively. After reaction with the disinfectant solution for 1 min, viral decreases of 3.98 ± 0.50, 3.58 ± 0.43, 4.12 ± 0.18, and 4.21 ± 0.13 log were identified at the front, rear, bottom, and sides, respectively. After reaction for 5 min, reductions by at least 4 log were shown at the front (4.36 ± 0.13 log), bottom (4.42 ± 0.33 log), and sides (4.96 ± 0.47 log), but not the rear (3.96 ± 0.31 log). After reaction for 10 min, reductions of at least 4 log were determined at all attachment locations, and the maximum degree of reduction was 5.54 ± 0.56 log (sides) while the minimum degree of reduction was 4.23 ± 0.25 log (rear) (Fig. 2B).

**Wall-type disinfection facility.** The passenger vehicle and large truck passed the wall-type disinfection facility at speeds of 3–5 km/h^-1^ while the disinfectant solution was being sprayed, and the reduction values as a function of the time (1, 5, 10 min) of reaction between the disinfectant solution and the virus were measured immediately after the vehicles passed the facility.

In the reaction between the virus on the carriers and the disinfectant solution for 1 min, reductions of at least 4 log were identified at all sections of the passenger vehicle, except for the rear. After reaction with the disinfectant solution for 1, 5, and 10 min, the virus at the front was reduced by at least 6 log (6.25 ± 0.94, 6.67 ± 0.52, and 7.18 ± 0.00 log, respectively) and no significant difference in reduction values was shown between the reaction times (*p* > 0.05). After reaction with the disinfectant solution for 1, 5, and 10 min, the virus at rear was reduced by 3.76 ± 0.28, 4.96 ± 0.15, and 6.48 ± 0.00 log, respectively, and significant differences occurred between the reaction times (1 vs. 5 min: *p* < 0.05; 1 vs. 10 min: *p* < 0.001). The viral reduction values at the bottom of the vehicle were 5.86 ± 0.20, 6.92 ± 0.16, and 7.01 ± 0.17, for 1, 5, and 10 min reaction with the disinfectant solution, respectively, and spraying for 5 and 10 min achieved more significant reductions than spraying for 1 min (*p* < 0.01). The resultant viral decreases at the sides of the vehicle were 5.68 ± 0.16 log (1 min spray), 6.23 ± 0.36 log (5 min spray), and 6.75± 0.25 log (10 min spray), with significant differences identified in the viral reduction values between 1 and 10 min reactions with the disinfectant solution (*p* < 0.05) (Fig. 2C).

After reaction with the disinfectant solution for 1 min, at least 4-log reductions of the virus were identified at all sections of the large truck, except for the rear. Reductions in the virus at the front (6.03 ± 0.13, 6.52 ± 0.66, and 7.18± 0.00 log), bottom (4.46 ± 0.33, 5.12 ± 0.30, and 5.83 ± 0.14 log) and sides (5.59 ± 0.69, 6.05 ± 0.73, and 6.69 ± 0.32 log) increased as the reaction time increased from 1 to 5, and 10 min, respectively. However, the rear of the vehicle did not present viral reduction values above 4 log, demonstrating corresponding reductions of 3.57 ± 0.42, 3.82 ± 0.34, and 3.98 ± 0.33 log after 1, 5, and 10 min. There was no significant difference in the viral reduction values detected on the sections of the large truck between the spray times (*p* > 0.05) (Fig. 2D).

### Correlation between Disinfectant Solution Coverage and AIV Reductions

**Fig. 3:**
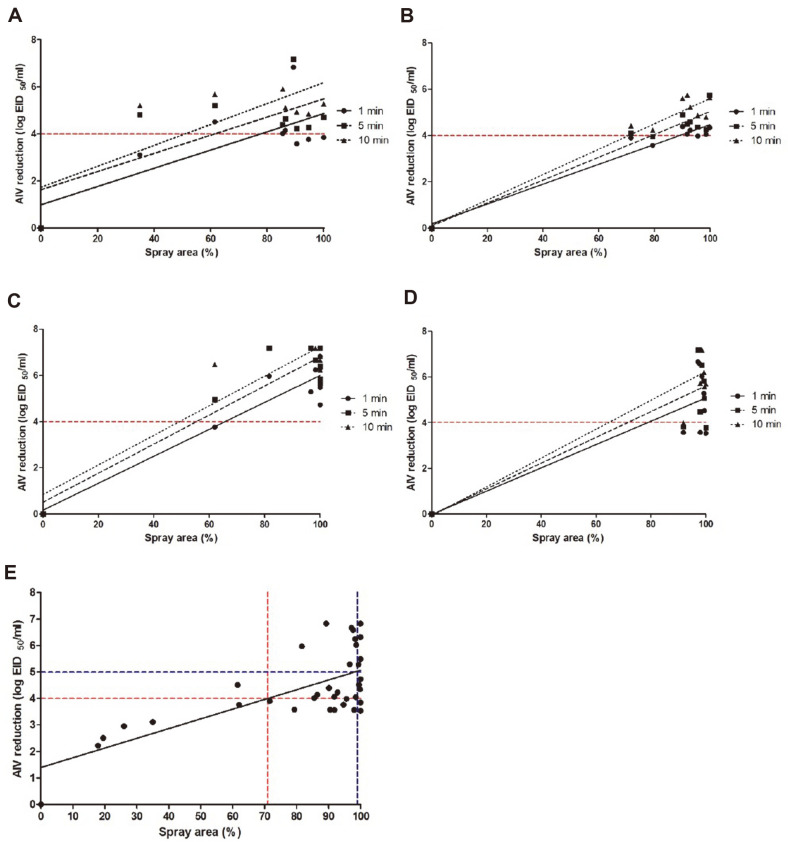
Correlation and linear regression results on disinfection facility. (**A**) Tunnel-type with passenger vehicle. (**B**) Tunnel-type with truck. (**C**) U-type with passenger vehicle. (**D**) U-type with truck. (**E**) Tunnel- and U-types with vehicles. Red: 4 log avian influenza virus (AIV) reduction; blue: 5 log AIV reduction.

**Correlation between physical and biological evaluations.** Correlation results in Table 2 show the relation between physical and biological evaluations. The passenger vehicle and large truck that passed through the tunnel-type disinfection facility showed viral reductions of at least 4 log when the vehicle surface coverage ratios were at least 78.0 and 89.9% (1 min), 62 and 79.8% (5 min), and 52 and 70.9% (10 min), respectively, after disinfection for 30 s. The corresponding values by treatment time were 0.7260 and 0.9265 (1 min), 0.4767 and 0.9217 (5 min), and 0.5700 and 0.9353 (10 min) (Figs. 3A and 3B, respectively).

For the surface of the passenger vehicle that passed the wall-type disinfection facility at speeds of 3–5 km/h, viral reductions by at least 4 log could be expected only when the disinfectant solution coverage ratios were at least 66% (1 min), 56% (5 min), and 50% (10 min). The R2 by treatment time were shown to be 0.8795 (1 min), 0.9597 (5 min), and 0.8533 (10 min) (Fig. 3C).

The surface of the truck that passed the wall-type disinfection facility at speeds of 3–5 km/h^-1^ demonstrated that disinfectant solution coverage ratios of at least 79% (1 min; R^2^ = 0.6388), 72% (5 min; R^2^ = 0.6702), and 65%(10 min; R^2^ = 0.8221) were necessary for a 4-log viral reduction (Fig. 3D).

To obtain the disinfectant solution coverage ratios necessary to reduce the virus existing on the outside of the vehicle by at least 4 log within 1 min, the coverage ratios and resultant AIV reductions of the passenger vehicle and the truck that passed through the disinfection facility were substituted into the horizontal axis and vertical axis, respectively. It was shown that to reach viral reduction values not less than 4 log, disinfectant solution coverage ratios of at least 71% should be achieved, regardless of vehicle size and disinfection facility type (Fig. 3E). In addition, where disinfectant solution coverage ratios of at least 99% were obtained at sections where pathogens exist, reductions of at least 5 log could be expected (R^2^ =0.4840; Table 3). Through the produced simple linear regression equation, the differences (residuals) between the observed and predicted AIV reductions varied between –0.95 and 1.76.

## Discussion

In the present study we intended to find the significance of the physical and biological performance evaluation methods for disinfection facilities by using WSPs and AIV, and examine the efficacy of these assessment strategies and any additional issues that must be considered.

Using two representative types of disinfection facilities that are already installed and operated at domestic livestock farms and related facilities, the disinfectant solution coverage ratios for accessing vehicles were measured, and the associated viral reduction values were established. For the purposes of the experiment, first, a method was developed to estimate the physical performance of the disinfection facilities, based on the disinfectant solution coverage for accessing vehicles. Next, biological evaluation methods and standards were prepared for appraisal of the pathogen removal performances. Finally, the relationship between the physical and biological evaluation results was identified, and the pathogen removal performances of the disinfection facilities could be indirectly assessed.

Although various factors, such as the components of disinfectants, workable concentrations and temperatures, and the presence of organic matter and surface materials, had to be considered to obtain appropriate disinfection and reduction effects on pathogens [14, 30, 31, 37], direct contact between the components of disinfectants and pathogens should happen first. In disinfection facilities, besides providing a certain minimal level of disinfectant solution coverage to the passing vehicle or object, the existence of disinfection blind spots should also be prevented for sections where pathogens may be present, such as the vehicle bottom and wheels.

By attaching WSPs to the front, rear, bottom, and sides of the vehicles, blind spots of the disinfection facilities and appropriate operating procedures could be examined. The tunnel-type disinfection facility showed significant differences in the resultant coverage of the bottom of the passenger vehicle and the truck between disinfectant solution spray times. When the disinfectant solution was sprayed for 60 s, the coverage ratios were at least 93% for both vehicles, but when the disinfectant solution was sprayed for 30 s, the bottom of the passenger vehicle had 74.10% coverage while the bottom of the truck showed 62.37% coverage. The sections included in the bottom were the wheels, mudguards, and vehicle chassis bottoms, and these areas had more complicated structures compared to the front, rear, and sides (handle, foothold, front door) of the vehicle.

The distance between the bottom of the passenger vehicle and the ground was 20 cm on average versus 20–33 cm at low regions and 90 cm at high regions, for the large trucks. The disinfectant solution is known to reach 1 m when the spray pressure is at least 0.96 MPa [27]. The spray pressure of the tunnel-type disinfection facility used in the study was adjusted to 4–8 MPa so that the disinfectant solution could reach the high regions of the truck bottom. It seems that adequate pressure, bottom nozzle spacing, and sufficient spray time are necessary for the disinfectant solution to be adequately applied to the wheels with complicated structures and the bottom of the vehicle chassis. Also, when the tunnel-type disinfection facility is operated, the disinfectant solution should be sprayed for at least 60 s.

The physical evaluation of the wall-type disinfection facility showed relatively high disinfectant solution coverage values compared to the tunnel-type disinfection facility. Disinfectant solution coverage of at least 90%was achieved on all sections of the large truck as well as all sections, except for the rear, of the passenger vehicle. It has been reported that the spraying rates are uniform when the nozzle diameter is 0.23–0.3 mm, and the resultant coverage for passing vehicles is highest when the pressure is at least 0.96 MPa or 20 kg/cm^2^ [25, 27, 28]. The spray pressure of the wall-type disinfection facility used in the present study was 3.5–6 MPa, and appropriate disinfectant solution coverage seems to have been achieved on all evaluated sections, including the vehicle bottom. Nevertheless, both the passenger vehicle and the truck showed the lowest coverage values at the rear, similarly to the results of Young-Il [9], which verified a lower coverage ratio for the vehicle rear than the front and sides. It appears that the disinfectant solution sprayed on moving vehicles could not be appropriately applied to the vertical rear of the vehicle because the solution flowed downward from top to bottom. Therefore, at places where the wall-type disinfection facility is operated, the vehicle driver should be informed to keep the entry speed at 3–5 km/h and not to raise the vehicle speed before completely passing through the disinfection facility, so that the entire vehicle can be sufficiently applied with the disinfectant solution.

For biological evaluation, a passenger vehicle and a large truck attached with dried carriers containing the virus were passed through the wall-type disinfection facility. As a result, reduction values not lower than 4 log could be identified on all evaluated sections after reaction with the disinfectant solution for 1 min. The viral reduction value at the rear of the passenger vehicle was 3.76 log (1 min), and the viral reduction value at the rear of the truck was 3.57 log (1 min). The relative low rear coverage ratios of the two vehicles (passenger vehicle: 61.98 ± 7.91%; truck: 91.86 ± 3.07%) compared to other parts appear to have influenced the AIV reduction values. It was shown that to obtain AIV reduction by 4 log after reaction with the disinfectant solution for 1 min, disinfectant solution coverage ratios of 66 and 79% are necessary for the passenger vehicle and large truck, respectively. These values seem to be associated with the AIV reduction value (3.76 log) of the passenger vehicle (R^2^ = 0.8795). In the large truck, the AIV reduction value (3.57 log) was lower compared to the coverage ratio, and this is considered attributable to the inability to control the outdoor environmental conditions (R^2^ = 0.6388).

Lower AIV reductions were observed for the vehicles that passed the tunnel-type (disinfection for 30 s) than the wall-type disinfection facility. Particularly, the vehicle bottom showed 0.3–1.5 log differences in proportion to the coverage ratio differences. Among the carrier attachment positions, the truck body bottom was the lowest section (20–33 cm). The maximum coverage ratio in this region was 88.02%, and the viral reduction value was 4.12 ± 0.18 log (1 min). At the rear, the coverage ratio was 79.35 ± 5.76%, and the viral reduction value was 3.58 ± 0.43 log (1 min). These results did not differ much from the coverage ratio necessary to obtain a viral reduction value of 4 log (1 min) for the large truck after passing through the tunnel-type disinfection facility, which was 89.9% (R^2^ = 0.9265). For the passenger vehicle, the coverage ratio required for 4-log reduction after 1 min reaction was 78%, and in this experiment, the viral reduction value of 4.51 ± 0.76 log was obtained at a coverage ratio of 74.10 ± 8.45%, similarly to the correlation value (R^2^ = 0.7260). Consequently, it appears that the disinfection nozzles should be operated for at least 60 s to achieve appropriate levels of coverage of all sections of the vehicle passing through the tunnel-type disinfection facility. These results suggest that disinfectant solution coverage ratios not lower than 71% are necessary to obtain minimum viral reduction values of 4 log, regardless of the types of disinfection facilities and vehicles, and at least a 5-log reduction can be expected when the coverage is at least 99%(R^2^ = 0.4840).

The main ingredient of the disinfectant used in the present study was citric acid, and the virus on the stainless-steel carriers was reduced by at least 4 log after the vehicle passed through the disinfection facility. Likewise, Jang *et al*. [37] indicated that pathogens on stainless-steel surfaces were reduced by a maximum 6 log after reaction for 5 min and by at least 4 log after reaction for 1 min. Since citric acid becomes less effective in the presence of organic matter, higher disinfection effects can be expected if disinfection against pathogens is conducted after removal of organic matter through high-pressure washing [14, 30, 32, 39]. Although relatively lower viral reduction values were established for the tunnel-type than wall-type disinfection facility, if the disinfection effect is increased in a shorter time, through the use of a more effective disinfecting component, or if physical removal of organic matter and pathogens is performed by an addition of high-pressure washing, additional reduction effects can be obtained.

This experiment is meaningful in that physical and biological disinfection facility performance evaluation methods were developed using actual-sized vehicles, and disinfection facilities and minimum disinfectant solution coverage standard and operation time necessary for proper disinfection were presented, through the analysis of the correlation between the two evaluation results.

If the developed evaluation methods are utilized, first, the physical and biological performance of disinfection facilities already installed and in operation can be ascertained. To date, the regulations, practices, and standards, for example, to assess disinfection facilities have not be prepared. Instead, there has been a tendency to focus on the public relations of installation companies and cost reduction rather than facility performance. However, the checking of facility performance and performance supplementation can be demanded and the minimum performance can be presented for new installations through the application of the evaluation methods. Second, the disinfection facility manufacturing market has problems, such as the fact that entry barriers are so low that many companies are not competitive, the low durability of products leads to high damage ratios, repair and maintenance services are not smoothly provided, and proper disinfection is not conducted. By applying the performance evaluation methods, the technology development of disinfection facility manufacturers can be encouraged and companies that are not competitive can be excluded from the market to induce the protection of animal husbandry and proper disinfection. Third, the awareness of disinfection by livestock industry workers and related personnel can be raised by presenting proper ways to use disinfection facilities and standards for disinfection. Given that livestock industry workers would greatly benefit from knowledge about the efficacy of actual disinfection procedures for disease prevention [40, 41], the results of the present study will contribute to the prevention and spread of disease introduction into domestic livestock farms and related facilities.

To effectively prevent further infectious diseases in South Korea, more studies are considered necessary on disinfection facility performance evaluation under diverse conditions in terms of temperatures, vehicle types (*e.g.*, feed and manure transport), disinfectant components, and disinfection methods (*e.g.*, ultraviolet irradiation, hot winds). Although the use of citric acid as a disinfectant has been reported to be effective in reducing the bacterial load [42, 43], there is a need to evaluate the efficacy of disinfection facilities in reducing the same.

## Figures and Tables

**Table 1:** Number of WSPs used per section.

	Passenger vehicle	Truck
Front	3	3
Rear	3	3
Bottom*	7	13
Side body**	7	7
Total	20	26

*Underparts: Underbody (9: L 3, M 3, H 3), Mudflap (2), Tire (2)

L: 20-33 cm, M: 50 cm, H: 90 cm

**Side body: Door or Side, Pedal, Handle

**Table 2:** Correlation and linear regression results.

Type		1 min	5 min	10 min
Tunnel	Passenger vehicle	y=0.03870x+0.990	y=0.0386x+1.624	y=0.04499x+2.225
	Pearson r	0.7367	0.6904	0.7550
	R^2^	0.7260	0.4767	0.5700
	4 log < (Spray)	78.0%	62.0%	52.0%
	Truck	y=0.04279x+0.1951	y+0.04925x+1.097	y=0.05471x+0.1283
	Pearson r	0.9729	0.9601	0.9671
	R^2^	0.9265	0.9217	0.9353
	4 log <(Spray)	89.9%	79.8%	70.9%
U	Passenger vehicle	y=0.05837x+0.1682	y=0.06297x+0.5081	y=0.06412x+0.0101
	Pearson r	0.9378	0.9272	0.9237
	R^2^	0.8795	0.8597	0.8533
	4 log < (Spray)	66.0%	56.0%	50.0%
	Truck	y=0.05102x-0.01887	y=0.05637x-0.02780	y=0.06321x-0.08169
	Pearson r	0.7992	0.8187	0.9067
	R^2^	0.6388	0.6702	0.8221
	4 log < (Spray)	79.0%	72.0%	65.0%

**Table 3:** Comparison of observed and predicted values of AIV reduction with spray results.

	Formula	Pearson r	R^2^	Spray (%)	

				4 log <	5 log <

1 min	y=0.03667x+1.1398	0.6957	0.4840	71	99

Coverage (%)	AIV reduction (log)			Residuals	

	Observed	Predicted			
100.00	6.32	5.06		1.2501	
100.00	5.49	5.06		0.4201	
99.99	6.83	5.06		1.7655	
99.85	4.35	5.06		-0.7144	
99.54	4.52	5.05		-0.5280	
99.33	5.28	5.04		0.2397	
98.63	6.03	5.01		1.0103	
98.50	4.05	5.01		-0.9599	
98.33	6.25	5.00		1.2413	
97.68	6.58	4.98		1.6002	
97.16	6.67	4.96		1.7042	
96.64	5.29	4.94		0.3483	
92.82	4.23	4.80		-0.5716	
91.76	4.07	4.76		-0.6977	
90.48	3.58	4.72		-1.1408	
90.12	4.40	4.70		-0.3076	
86.46	4.15	4.57		-0.4234	
85.51	4.02	4.53		-0.5186	
81.67	5.97	4.39		1.5772	
79.35	3.58	4.31		-0.7277	
71.58	3.90	4.02		-0.1228	
61.98	3.76	3.67		0.0893	
61.53	4.51	3.65		0.8558	
34.99	3.11	2.68		0.4290	
26.00	2.95	2.35		0.5986	
19.46	2.51	2.11		0.3984	
17.86	2.22	2.05		0.1671	
